# The Iron-Klotho-VDR Axis Is a Major Determinant of Proximal Convoluted Tubule Injury in Haptoglobin 2-2 Genotype Diabetic Nephropathy Patients and Mice

**DOI:** 10.1155/2018/7163652

**Published:** 2018-09-03

**Authors:** Inbal Dahan, Nadia Thawho, Evgeny Farber, Nakhoul Nakhoul, Rabea Asleh, Andrew P. Levy, Yan Chun Li, Ofer Ben-Izhak, Farid Nakhoul

**Affiliations:** ^1^Diabetes and Metabolism Lab, The Baruch Padeh Medical Center, Poriya, Lower Galilee, Israel; ^2^Nephrology and Hypertension Division, The Baruch Padeh Medical Center, Poriya, Lower Galilee, Israel; ^3^The Azrieli Faculty of Medicine in Zfat in the Galilee, Bar-Ilan University, Ramat-Gan, Israel; ^4^The Vascular Medicine Lab, Technion, Faculty of Medicine, Rappaport Institute, Haifa, Israel; ^5^Department of Medicine, The University of Chicago, Chicago, IL 60637, USA; ^6^Department of Pathology, Rambam Health Care Campus, Haifa, Israel; ^7^Technion, Haifa, Israel

## Abstract

The haptoglobin (Hp) genotype (1-1 and 2-2) is a major determinant of nephropathy progression in diabetes mellitus patients. Hp 2-2 diabetic mice have impaired Hb clearance and increased iron deposits and oxidative stress in the proximal tubules (PCT), leading to increased renal injury. However, the precise mechanism of the PCT injury in diabetic nephropathy (DN) remains elusive. In the kidney, 1,25(OH)_2_D3 suppresses the inflammatory response to renal tubular injury and requires normal renal expression of the *α*-klotho protein. In this study, we set out to test the hypothesis that the increased renal iron deposits in the PCT of Hp 2-2 DN affect the *α*-klotho-vitamin D receptor (VDR) axis and thereby exacerbates the PCT injury generated by the iron deposits. Immunohistochemical analysis of human and mouse kidney biopsies along with western blot analysis showed that the increased iron deposits in the PCT of the Hp 2-2 genotype were accompanied with significantly decreased *α*-klotho and VDR renal expression but significantly increased 1-*α*-hydroxylase renal expression. In conclusion, the iron-klotho-VDR axis is a major player in the mechanism contributing to iron-mediated PCT injury in diabetic Hp 2-2 mice and patients. Targeting this axis may open the way for new ideas regarding the pathogenesis and treatment of DN.

## 1. Introduction

Haptoglobin (Hp) is an acute phase protein that acts as an antioxidant by virtue of its ability to bind free hemoglobin (Hb) and prevent heme-iron–mediated oxidation accompanied by tissue damage [1, 2]. Two classes of Hp alleles are known (1 and 2). While the Hp 1 allele is found in all animal species, the Hp 2 allele is present only in humans. The Hp genotype is a major determinant of progression of diabetic nephropathy (DN) [3–5]. The Hp 1 protein is superior to the Hp 2 protein in binding to free Hb and neutralizing its oxidative potential [6–8]. Moreover, Hp 2-2 is significantly associated with the development of reduced glomerular filtration rate (GFR) and end-stage renal disease [5] and is associated with greater incidence of cardiovascular disease compared with Hp 1-1 [9–11]. The difference between the Hp types is exaggerated in the diabetic state as extracorpuscular Hb is increased due to increased red cell fragility, and the ability of Hp to block the oxidative activity of Hb is impaired when Hb becomes glycated [1, 7, 8, 12]. Previously, we reported on the ability to recapitulate the interaction between the Hp genotype and DM in DN mice [13]. This mouse model represents DN with the spectrum of micro- and macrovascular complications similar to the human disease [13]. Using this mouse model, we have demonstrated marked differences in renal structure and function between the Hp 1-1 and Hp 2-2 DM mice; Hp 2-2 DM mice had a significant increase of iron deposits in the lysosomes of the kidney proximal convoluted tubule cells (PCT) with lysosomal and renal damage as well as increased features of glomerular disease characteristic of early human DN [7, 13, 14].

*Klotho* is a novel antiaging gene encoding a protein with multiple pleiotropic effects [15]. The klotho family of proteins consists of three members: *α*-klotho, *β*-klotho, and *γ*-klotho. The *α-klotho* gene is highly expressed in the distal and proximal convoluted tubular epitheliums of normal adult kidneys [16] and is affected by pathophysiological conditions including long-term hypertension, oxidative stress, diabetes, and chronic renal failure [16–21]. *α*-Klotho protein exists in two forms: a single pass transmembrane protein and a secreted protein [21]. The secreted form is involved in important biological processes including suppression of oxidative stress [15, 21]. The transmembrane *α*-klotho functions as a coreceptor for FGF-23, a bone-derived hormone that suppresses phosphate reabsorption and 1,25(OH)_2_D3 biosynthesis in the kidney via 1-*α*-hydroxylase [22]. 1,25(OH)_2_D3 stimulates both *α*-klotho and FGF-23, and both FGF-23 and *α*-klotho inhibit 1,25(OH)_2_D3 [23].

The involvement of vitamin D in kidney injury has been extensively studied. Vitamin D is transported to the liver, where it is first hydroxylated in position 25 to yield 25-hydroxyvitamin D. 25-Hydroxyvitamin D is further hydroxylated by 1-*α*-hydroxylase in the PCT of the kidney, to yield its active form 1,25(OH)_2_D3 [24]. The active 1,25(OH)_2_D3 binds to the intracellular vitamin D receptor (VDR) to activate vitamin D response elements within target genes [24, 25]. In the kidney, vitamin D is important for maintaining podocyte health, preventing epithelial to mesenchymal transformation, and suppressing renin gene expression and inflammation [26, 27]. Furthermore, in chronic kidney disease (CKD) patients, as renal function declines, serum levels of 1,25(OH)_2_D3 progressively decrease due to PCT injury, leading to a vitamin D-deficient state [27]. However, the precise mechanism of the PCT injury in DN with Hp 2-2 genotype remains elusive and needs to be determined. In this study, we set out to test the hypothesis that the increased iron deposits in the PCT of Hp 2-2 DN mice and patients affect the *α*-klotho-vitamin D receptor axis, leading to more prominent renal damage and a vitamin D-deficient state.

## 2. Materials and Method

### 2.1. Study Design

The current study was carried out to explore the involvement of the iron-klotho-VDR axis in the renal PCT injury of DN mice and patients with the Hp 2-2 genotype compared with Hp 1-1 genotype. The study includes experiments with the genetically engineered mouse model of DN with the Hp 2-2 genotype as well as type 2 diabetic (DM) and nondiabetic (nonDM) CKD patients at different stages (1–4) from The Baruch Padeh Medical Center, Poriya.

Kidney biopsies from patients and mice with different Hp genotypes (2-2 and 1-1) were subject to immunohistochemistry (IHC), and the mice kidney lysates were subjected to western blot analysis.

### 2.2. Animal Studies

All procedures were approved by the Animal Care Committee of the Technion (protocol number IL-112-11-11). We used our Hp mice model that we used in our previous study [13, 14]. The Hp 2-2 DM mice is a good model for DN since it has increased features of glomerular disease characteristic of early human DN including hyperfiltration, glomerular hypertrophy, and albuminuria [13]. All mice were of C57B1/6 genetic background. The Hp 2 allele is present only in humans. All other species have only an Hp 1 allele, which is highly homologous with the human Hp 1 allele. Thus, wild-type mice carry the Hp 1 allele (referred to herein as Hp 1-1 mice). The construction of the murine Hp 2 allele and the targeting of its insertion by homologous recombination to the murine Hp genetic locus have been previously described [2]. Each group (Hp 2-2 and Hp 1-1 DM and nonDM) included 3 mice. Seven-week-old male mice were made diabetic by intraperitoneal injection of 50 mg/kg of STZ per day freshly prepared in 10 mM citrate buffer (pH 4.2) for 5 consecutive days. Mice were fed normal chow and exposed to a 12 : 12-hour light-dark cycle. Mice were sacrificed after a DM duration of 2 months (nonDM mice and DM mice were sacrificed at the same age). There were no differences in blood spot glucose levels between mice with the different Hp genotypes. After mice were sacrificed, the kidneys were removed, washed in saline, and either fixed in formalin for morphometric and immunohistological analysis [2] or placed in liquid nitrogen for western blot analysis and biochemical studies.

### 2.3. Patients

All experiments were approved by the Helsinki Committee of Poriya Hospital, and informed consent was obtained from all participants. The study included 42 biopsies from type 2 DM and nonDM CKD patients (males and females), above 18 years old from our nephrology outpatient clinic in Poriya Medical Center. Excluded from the study were oncology patients and pregnant women. The human kidney biopsies were done only for clinical reasons, but after patient consent, we used them for this study as well. For this study, we used only the renal biopsies from Hp 1-1 and 2-2 genotypes. Twenty-two biopsies were from CKD type 2 DM patients, and 20 biopsies from CKD nonDM patients served as controls. All the study participants were screened for the Hp genotype. Demographic and clinical data concerning the patients (age, sex, diabetic status, comorbidities, and medications used) were obtained directly from the patients or from their medical files.

### 2.4. Haptoglobin Typing

Hp typing of the study participants was performed on plasma samples by polyacrylamide gel electrophoresis as previously described [28]. In brief, 10 *μ*L of Hb-enriched plasma was subjected to electrophoresis in a nondenaturing gel, and the gel was subsequently immersed in a solution containing a congener of benzidine with a precipitate forming in the gel corresponding to the location of Hb-Hp complexes. The Hp type of the sample was determined by the banding pattern of the Hp-Hb complexes; each of the three Hp types has a characteristic banding fingerprint.

### 2.5. Western Blot Analysis

Mice renal tissues were homogenized with a protein lysis buffer containing protease and phosphatase inhibitors. The protein content of lysates was determined by the Bradford assay, and equal amounts of protein were boiled in a loading buffer and analyzed by SDS-PAGE. Gels were transferred to Protran membranes (Whatman), blocked with 5% BSA in TBS-T, incubated with a primary antibody in 5% BSA in TBS-T at 4°C overnight, then washed, and incubated with the HRP-conjugated secondary antibody (1 : 7500, Jackson) in TBS-T for 45 minutes at RT. Bands were visualized using EZ-ECL Chemiluminescence Detection Kit (Biological Industries), and the intensities of the bands were analyzed using Fujifilm ImageGaugeV software. Primary antibodies for klotho (1 : 2000; LSBio), *β*-actin (1 : 5000; Sigma), GAPDH (1 : 7500; Abcam), VDR (1 : 750; Novus Biologicals), and 1-*α*-hydroxylase (CYP27B1 (1 : 750; Abcam)) were used; secondary antibodies were HRP-linked goat anti-mouse or goat anti-rabbit (1 : 5000, Jackson).

### 2.6. Immunohistochemistry (IHC)

Renal tissues from mice and human biopsies were fixed in 4% formaldehyde for 24 hours, paraffin embedded, and sectioned at 5 *μ*m for immunostaining. Sections were deparaffinized in xylene and graded alcohols, followed by antigen retrieval and endogenous peroxidase quenched by H_2_O_2_. Sections were then incubated overnight at 4°C with primary antibodies at the following dilutions in blocking solution (CAS-block, Invitrogen): klotho (1 : 400; LSBio), VDR (1 : 200; Novus Biologicals) and 1-*α*-hydroxylase (CYP27B1 (1 : 200; Abcam)). HRP-polymer anti-rabbit (Nichirei and Dako) was used as a secondary antibody. Sections were developed with DAB Chromogen (Thermo Scientific). Slides were counterstained with Mayer's Hematoxylin, dehydrated, and mounted with Entellan (Merck). Images were obtained at 20x and 10x using an Axio Lab.A1 microscope with the Axiocam 105 color digital camera and ZEN software. Exposure times were kept constant for all samples. Quantification of the IHC staining was performed by using Panoramic Viewer and Image-Pro software version 7.

### 2.7. PAS Staining

Slides were deparaffinized, rehydrated, and then incubated in 2% periodic acid for 15 minutes followed by Schiff's reagent (Merck Millipore) for 25 minutes. Counter-staining was performed with filtered hematoxylin.

### 2.8. Iron Staining by Modified Perls' DAB Staining

After deparaffinization in xylene and rehydration through graded ethanol, sections from mice and human biopsies were washed in PBS and incubated in a peroxidase blocking solution of 3% H_2_O_2_ for 10 min. Then the slides were treated in Prussian blue solution for 20 min, washed with PBS, and subsequently incubated with 3,3'-DAB and 2 *μ*L of 30% H_2_O_2_ DAB (Sigma) for 20 min in a dark humidified chamber. Then slides were counterstained with Mayer's Hematoxylin, dehydrated, and mounted with Entellan (Merck).

### 2.9. Statistics and Quantification

IHC staining was quantified by Panoramic Viewer and Image-Pro (version 7) software according to the Hot-spot method with the most stained fields in each Hp group accompanied with 95% confidence interval analysis. PAS staining was conducted to ensure that the number of tubules in the different renal sections was similar during the IHC quantification. Positively stained tubules were manually counted within the area of the whole field at optical magnification 10x. The quantification was based on at least 10 fields in each group and included the percentage of the total stained area. The total stained area was calculated based on the ratio of the manually selected stained area to the total area of the field. All manual procedures were performed without knowledge of mouse or patient background.

All results are reported as mean ± SEM. Comparison between groups was performed using 2-tailed Student's *t*-test, ANOVA, Kruskal-Wallis test, and Tukey's LSD method for pairwise comparisons, with a *p* value < 0.05 considered statistically significant. Data analysis was done using IBM SPSS software version 20.0.

## 3. Results

The Hp protein products of the Hp 1 and Hp 2 alleles differ in both their biochemical and their functional properties such as antioxidant ability and Hb clearance from the plasmatic compartment, and these differences are exaggerated in diabetes [6–8]. Hence, in the current study, we stratified the study participants according to their Hp genotype and their diabetes status and explored the involvement of the iron-klotho-VDR axis in the renal PCT injury. The study participants' characteristics are presented in Table 1. Most of our CKD patients (83%, *n* = 35) have the Hp 2-2 genotype and had reduced GFR levels compared with Hp 1-1 genotype. Sixty percent (*n* = 21) of these patients are diabetic nephropathy patients (Table 1). This distribution is in agreement with previous studies that have shown that Hp 2-2 genotype is associated with the development of reduced GFR and increased development of diabetic complications [5–8].

### 3.1. Increased Iron-Rich Deposits in the PCT Cells of DN Mice and Patients with Hp 2-2 Genotype

**Figure 1:**
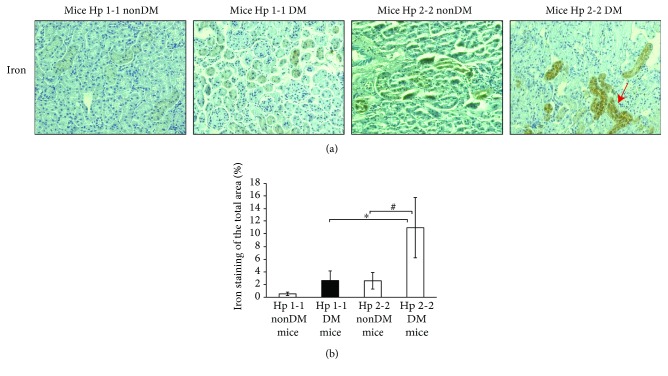
Iron staining in Hp 2-2 and Hp 1-1 renal sections of diabetic nephropathy and nondiabetic mice. (a) There was a significant increase of iron deposits represented by the DAB-enhanced Prussian blue staining (brown, indicated by a red arrow) in the proximal convoluted tubule (PCT) of Hp 2-2 diabetic (DM) sections compared with diabetic Hp 1-1 or with Hp 2-2 nondiabetic (nonDM) sections. (b) Quantification of the iron staining shows a significant increase in the percentage of the iron-stained area of Hp 2-2 DM mice compared with DN Hp 1-1 mice (^∗^*p* < 0.001) or compared with nonDM Hp 2-2 mice (^#^*p* < 0.001).

We previously demonstrated an increased accumulation of iron deposits in the renal PCT of Hp 2-2 DN mice [7, 13, 14]. To confirm the prevalence of renal iron deposits in our Hp 2-2 DN mice, renal samples of mice with Hp 1-1 and Hp 2-2 genotypes were stained for iron by using DAB staining. As shown in Figures 1(a) and 1(b), there was a 4-fold increase in the percentage of the iron-stained area in PCT of Hp 2-2 DM mice (11 ± 4.74%) compared with Hp 1-1 DM (2.71 ± 1.45%, *p* < 0.001) or with Hp 2-2 nonDM mice (2.59 ± 1.33%, *p* < 0.001).

**Figure 2:**
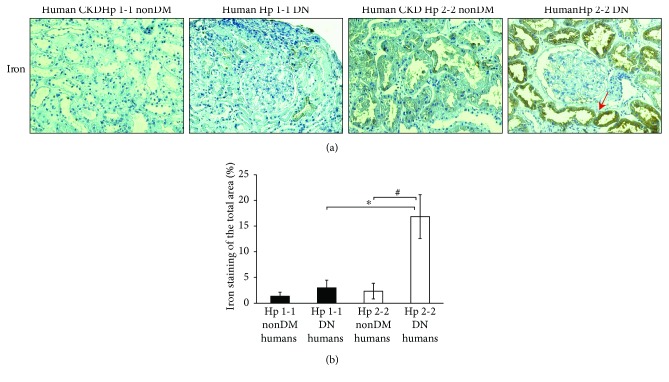
Iron staining in Hp 2-2 and Hp 1-1 renal sections of diabetic nephropathy and nondiabetic CKD patients. (a) There was a significant increase of iron deposits represented by the DAB-enhanced Prussian blue staining (brown, indicated by a red arrow) in the PCT of Hp 2-2 diabetic nephropathy (DN) patients compared with DN Hp 1-1 or Hp 2-2 CKD nondiabetic (nonDM) patients. (b) Quantification of the iron staining shows a significant increase in the percentage of the iron-stained area of Hp 2-2 DM patients compared with the DN Hp 1-1 genotype (^∗^*p* < 0.001) or compared with the nonDM CKD Hp 2-2 genotype (^#^*p* < 0.001).

To further investigate the prevalence of renal iron deposits also in Hp 2-2 DN patients, renal samples of CKD patients with Hp 1-1 and 2-2 genotypes were stained for iron. There was nearly a 5-fold increase in the iron-stained area in Hp 2-2 DN renal sections (16.86 ± 4.25%) compared with DM Hp 1-1 sections (3.03 ± 1.48%, *p* < 0.001) or compared with nonDM CKD Hp 2-2 sections (2.36 ± 1.51%, *p* < 0.001, Figures 2(a) and 2(b)).

### 3.2. Decreased Renal *α*-Klotho Expression in DN Mice and Patients with Hp 2-2 Genotype

**Figure 3:**
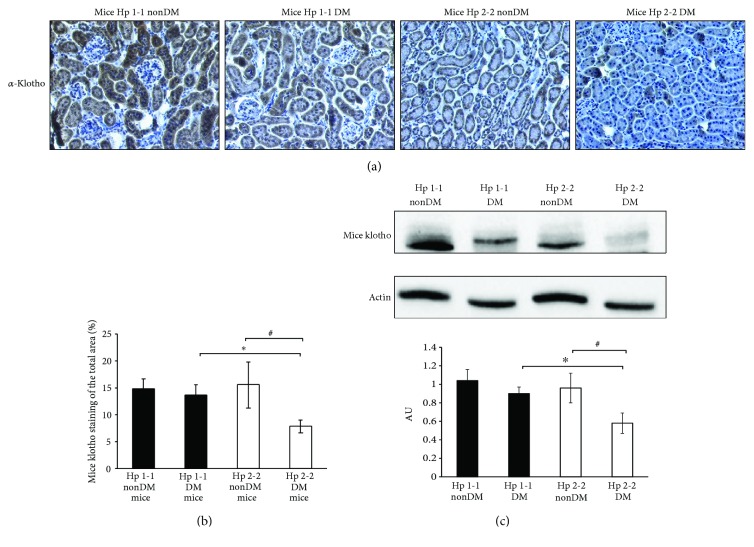
(a) *α*-Klotho renal expression in nondiabetic and diabetic nephropathy mice. (b) The percentage of the *α*-klotho-stained area is significantly decreased in the renal tubules of Hp 2-2 diabetic (DM) sections compared with Hp 1-1 diabetic (^∗^*p* < 0.05) or with nondiabetic (nonDM) Hp 2-2 sections (^#^*p* < 0.05). (c) *α*-Klotho expression in mice renal lysates from Hp 1-1 and Hp 2-2 nondiabetic (nonDM) and diabetic (DM) mice. Proteins were detected by immunoblotting with antibodies against *α*-klotho and actin. The values represent the mean ± SEM for 3 independent experiments subjected to 2-tailed, 2-sampled unequal variance Student's *t*-test, with ^#^*p* < 0.05 compared with Hp 2-2 nonDM and ^∗^*p* < 0.05 compared with Hp 1-1 DM. AU: arbitrary units.

Previous studies have indicated that renal *α-klotho* gene expression is regulated by pathophysiological conditions including long-term hypertension, oxidative stress, diabetes, and chronic renal failure in both animal models and humans [16, 17, 20, 29–32]. Hence, we evaluated whether the increased renal iron deposits associated with the Hp 2-2 genotype also affect the *α*-klotho renal expression both in DN mice and patients. As shown in Figures 3(a) and 3(b), the percentage of the *α*-klotho-stained area was significantly reduced in the tubules of Hp 2-2 DN mice (7.8 ± 1.22%) compared with DM Hp 1-1 genotype (13.58 ± 2.01%, *p* < 0.05) or with nonDM Hp 2-2 genotype (15.53 ± 4.2%, *p* < 0.05).

In order to strengthen these results, western blot analysis was done using the mice renal lysate (Figure 3(c)). *α*-Klotho expression was significantly decreased in Hp 2-2 DN mice (0.58 ± 0.11 arbitrary units (AU)) compared with DM Hp 1-1 genotype (0.9 ± 0.07 AU, *p* < 0.05) or with Hp 2-2 nonDM mice (0.96 ± 0.16 AU; *p* < 0.05).

**Figure 4:**
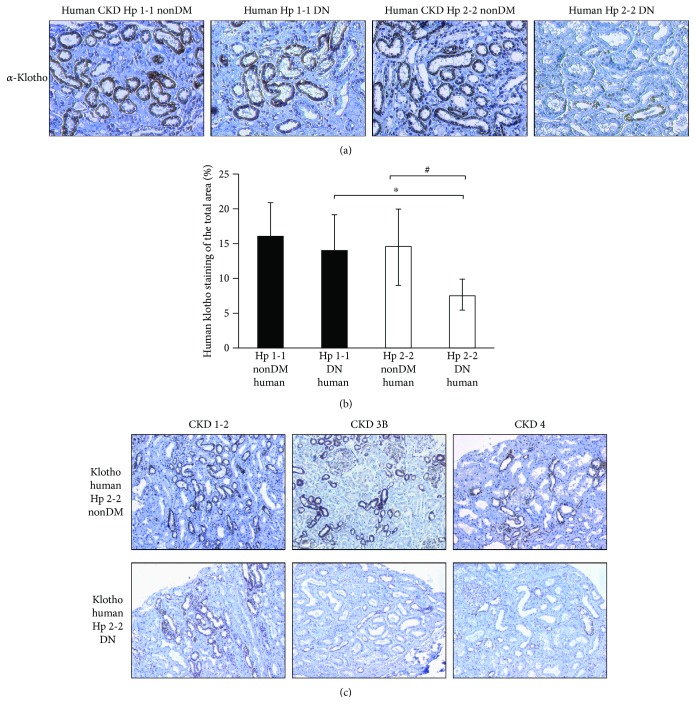
(a) *α*-Klotho renal expression in nondiabetic and diabetic nephropathy patients. (b) The percentage of the stained area of *α*-klotho is significantly decreased in the renal tubules of diabetic (DM) biopsies with Hp 2-2 genotype compared with Hp 1-1 diabetic (^∗^*p* < 0.05) or with nondiabetic (nonDM) Hp 2-2 genotype (^#^*p* < 0.05). (c) In chronic kidney disease (CKD) patients with the Hp 2-2 genotype, the renal expression of *α*-klotho declines with the progression in the CKD stage, and it is exacerbated in the diabetic state.

Similar to the results obtained in mice, the percentage of the stained area of renal *α*-klotho was also reduced by nearly 2-fold in tubules of Hp 2-2 DN patients (7.5 ± 2.36%) compared with DM Hp 1-1 genotype (14.02 ± 5.1%, *p* < 0.05) or with CKD nonDM Hp 2-2 genotype (14.58 ± 5.34%, *p* < 0.05, Figures 4(a) and 4(b)).

We further investigated whether the decline in renal function among Hp 2-2 CKD patients was associated with an exacerbated decrease in renal *α*-klotho expression. As shown in Figure 4(c), the renal *α*-klotho expression was gradually decreased along with the progression in CKD stages, and this decrease was exacerbated in diabetes.

### 3.3. Decreased Expression of Renal Vitamin D Receptor (VDR) and Increased Expression of 1-*α*-Hydroxylase in DN Hp 2-2 Mice and Patients

**Figure 5:**
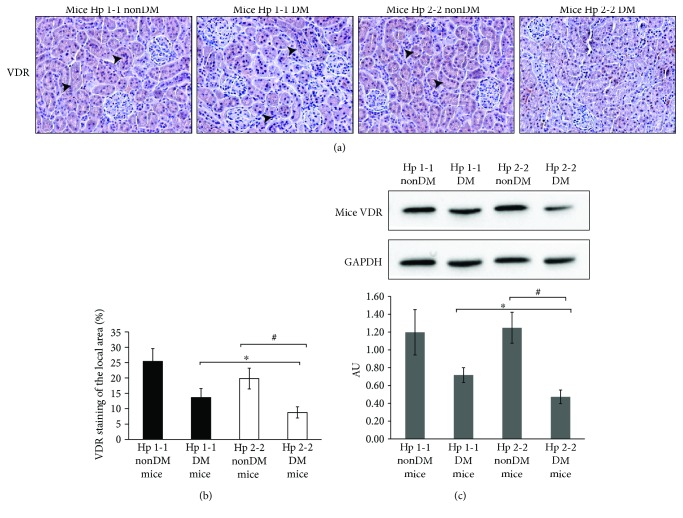
Renal vitamin D receptor (VDR) expression in nondiabetic (nonDM) and diabetic (DN) mice. (a) Renal sections from Hp 1-1 and 2-2 mice were subjected to IHC of VDR (brown, indicated by arrowheads). (b) There was a significant decrease in the percentage of the VDR-stained area in the tubules of Hp 2-2 DM renal sections compared with Hp 1-1 DM (^∗^*p* < 0.05) or with nonDM Hp 2-2 renal sections (^#^*p* < 0.05). (c) Western blot analysis of VDR with the mice renal lysate. Proteins were detected by immunoblotting with antibodies against VDR and GAPDH. The values represent the mean ± SEM for 3 independent experiments subjected to 2-tailed, 2-sampled unequal variance Student's *t*-test, with ^∗^*p* = 0.001 compared with Hp 2-2 nonDM and ^#^*p* = 0.005 compared with Hp 1-1DM. AU: arbitrary units.

Normal expression of renal *α*-klotho is required for normal vitamin D homeostasis via downregulation of 1-*α*-hydroxylase and upregulation of 24-hydroxylase. Therefore, we tested whether the decrease in *α*-klotho renal expression shown in the Hp 2-2 DN genotype was accompanied with altered expression of renal VDR and 1-*α*-hydroxylase. As shown in Figures 5(a) and 5(b), the percentage of the VDR-stained area in mice renal sections was significantly decreased in the PCT of DN Hp 2-2 mice (8.3 ± 1.83%, *p* < 0.05) compared with DM Hp 1-1 mice (13.6 ± 2.9%) or with Hp 2-2 nonDM mice (19.8 ± 3.4%, *p* < 0.05).

Western blot analysis of the mice renal lysates (Figure 5(c)) strengthened these results, as VDR expression was significantly decreased in Hp 2-2 DN mice (0.47 ± 0.08 AU) compared with Hp 1-1 DM mice (0.72 ± 0.08 AU; *p* = 0.005) or with Hp 2-2 nonDM mice (1.25 ± 0.17 AU; *p* = 0.001).

**Figure 6:**
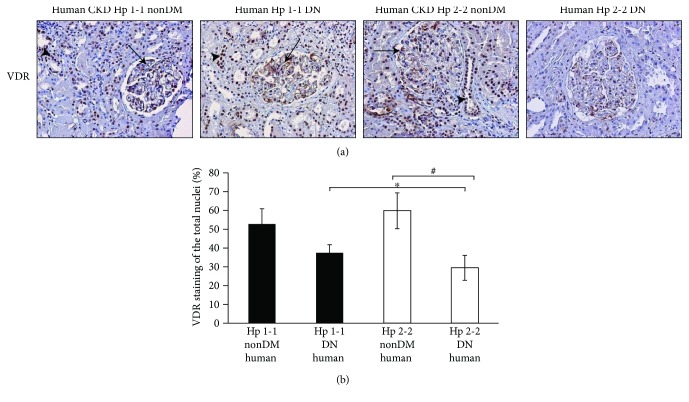
Renal vitamin D receptor (VDR) expression in nondiabetic (nonDM) and diabetic (DN) patients. (a) Renal biopsies from Hp 1-1 and 2-2 CKD patients were subjected to IHC of VDR. There was a significant decrease in VDR expression (brown) in the tubules (indicated by arrowheads) and in the glomerulus (indicated by black arrows) of Hp 2-2 DM renal sections compared with Hp 1-1 DM or with nonDM Hp 2-2 renal sections. (b) Quantification of VDR staining shows a significant decrease in the percentage of stained nuclei of Hp 2-2 DM sections compared with DM Hp 1-1 sections (^∗^*p* < 0.001) or compared with the nonDM Hp 2-2 genotype (^#^*p* < 0.001).

The renal expression of VDR was decreased also in human's renal sections. VDR staining in human's sections was mainly in the nucleus. As shown in Figures 6(a) and 6(b), the percentage of stained nuclei significantly decreased in CKD Hp 2-2 DN renal sections (29.5 ± 6.61%) compared with DM Hp 1-1 sections (37.44 ± 4.35%, *p* < 0.001) or compared with nonDM Hp 2-2 sections (59.9 ± 9.55%, *p* < 0.001).

**Figure 7:**
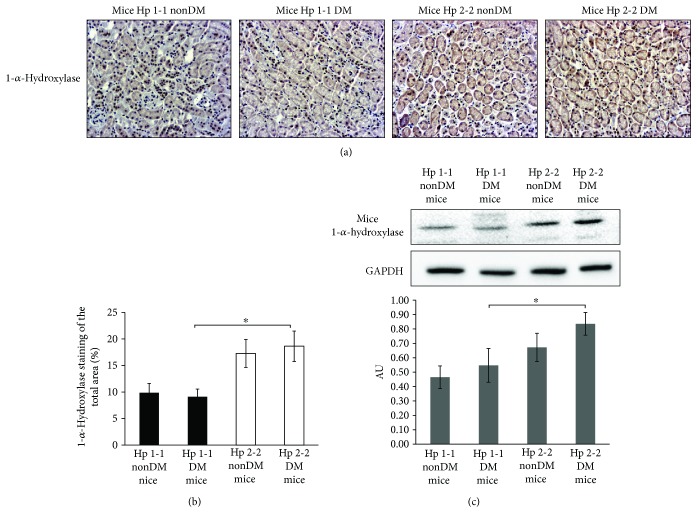
1-*α*-Hydroxylase expression in diabetic and nondiabetic (nonDM) mice. (a) Slides from mice renal biopsies were subjected to IHC of 1-*α*-hydroxylase. (b) There was a significant increase in the percentage of the stained area of 1-*α*-hydroxylase in the tubules of Hp 2-2 DM renal sections compared with Hp 1-1 DM sections (^∗^*p* < 0.05) but insignificant compared with nonDM Hp 2-2 genotype. (c) Western blot analysis of 1-*α*-hydroxylase with the mice renal lysate. Proteins were detected by immunoblotting with antibodies against 1-*α*-hydroxylase and GAPDH. The values represent the mean ± SEM for 3 independent experiments subjected to 2-tailed, 2-sampled unequal variance Student's *t*-test, with ^#^*p* = 0.008 compared with Hp 1-1DM. AU: arbitrary units.

**Figure 8:**
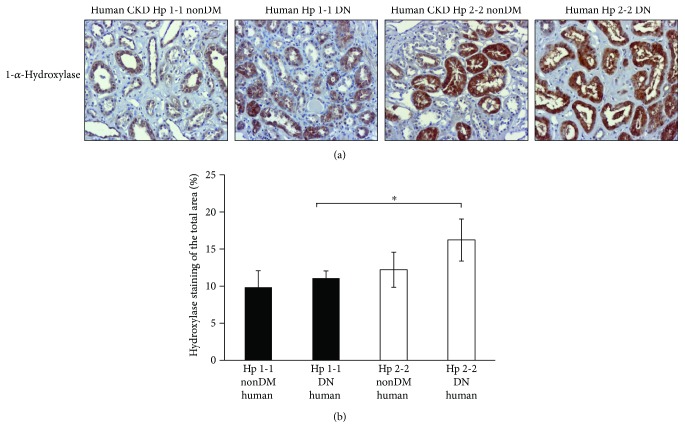
1-*α*-Hydroxylase expression in diabetic and nondiabetic (nonDM) CKD patients. (a) Slides from human's renal biopsies were subjected to IHC of 1-*α*-hydroxylase. (b) There was a significant increase in the percentage of the stained area of 1-*α*-hydroxylase in the tubules of Hp 2-2 DM renal sections compared with Hp 1-1 DM sections (^∗^*p* < 0.05) but insignificant compared with the nonDM Hp 2-2 genotype.

Contrary to the decreased expression of VDR that was observed in DM mice and in patients with Hp 2-2 genotype, renal expression of 1-*α*-hydroxylase was increased in these mice and patients (Figures 7(a) and 8(a)). 1-*α*-Hydroxylase staining in mice showed a significant decrease in the percentage of the stained area of 1-*α*-hydroxylase in DN Hp 2-2 mice (18.64 ± 2.87%) compared with DM Hp 1-1 sections (9.1 ± 1.45%, *p* < 0.05) but insignificant compared with nonDM Hp 2-2 sections (17.26 ± 2.62%, *p* = 0.611, Figure 7(b)).

Western blot analysis with the mice renal lysates (Figure 7(c)) supports these observations. As shown in Figure 7(c), there was a significant increase in 1-*α*-hydroxylase expression in Hp 2-2 DN mice (0.84 ± 0.08 AU) compared with DM Hp 1-1 genotype (0.55 ± 0.12 AU, *p* = 0.008) but insignificant compared with Hp 2-2 nonDM mice (0.67 ± 0.1 AU).

Similar to mice results, 1-*α*-hydroxylase staining in human renal sections also showed a significant decrease in the percentage of the stained area in DN Hp 2-2 sections (16.22 ± 2.84%) compared with DM Hp 1-1 sections (11 ± 0.98%, *p* < 0.05) but insignificant compared with nonDM Hp 2-2 sections (12.21 ± 2.37%, *p* = 0.67, Figures 8(a) and 8(b)).

## 4. Discussion

Oxidative stress is an important possible causative factor of DN [33]. Hence, the Hp genotype is a major determinant of DN progression [3–5]. The Hp 2 protein is inferior to the Hp 1 protein in binding to free Hb and neutralizing its oxidative potential [6–8], and this difference is exaggerated in the diabetic state. We have previously demonstrated a correlation between the Hp 2-2 genotype and the accumulation of iron deposits in the lysosomes of the renal PCT in mice, which results in lysosomal membrane injury and renal cells damage caused by oxidative stress [13, 14]. The source of excess PCT iron in Hp 2-2 DN is Hb [8]. In Hp 2-2 DM, the normal clearance mechanism via the CD163 receptor for the Hp-Hb complex is severely impaired with approximately a 5-fold increase in the half-life of the complex [14, 34]. Furthermore, iron is known to enhance organ damage induced by oxidants to generate highly toxic hydroxyl radicals. Thus, it is possible that the deposition of renal iron leads to a decrease in *α*-klotho renal expression by inducing oxidative stress [35, 36].

In this study, we set out to explore the hypothesis that the excess iron deposition in DN individuals and mice with Hp 2-2 genotype leads to downstream effects on the *α*-klotho-VDR axis that consequently leads to renal PCT injury. We have shown significantly increased iron deposits in the PCT of Hp 2-2 DN mice and patients that were associated with decreased *α*-klotho and VDR renal expression. We further found out that in the Hp 2-2 genotype, *α*-klotho renal expression is gradually reduced with the progression in CKD stages, and this decrease is more prominent in the diabetic state. This association is in agreement with previous studies that have shown that renal *α-klotho* gene expression is regulated by pathophysiological conditions including hypertension, diabetes, and chronic renal failure in animal models and in humans [16, 17, 20, 29–32].

It is known that CKD patients suffer from a vitamin D-deficient state, but the precise mechanism of the renal injury and the impairment of vitamin D activation remain elusive. Since normal renal expression of the *α*-klotho protein is required for normal 1,25-dihyroxyvitamin D homeostasis via downregulation of 1-*α*-hydroxylase and upregulation of 24-hydroxylase [37], we further explored whether the decreased *α*-klotho renal expression in Hp 2-2 DN genotype affects the expression of renal VDR and 1-*α*-hydroxylase. We found out that in DN Hp 2-2 patients and mice, while the expression of VDR was decreased, 1-*α*-hydroxylase renal expression was increased probably as a compensation mechanism.

In the kidney, active vitamin D is important for maintaining podocyte integrity and suppressing renin gene expression and inflammation [38]. Therefore, vitamin D has the potential to have a favorable impact on DN severity in Hp 2-2 phenotype via kidney protection from oxidative stress injury [39–41]. The beneficial effects of vitamin D on renal fibrosis in DN are mediated by VDR via restoration of *α*-klotho expression. Furthermore, *α*-klotho deficiency in combination with hyperglycemia can aggravate the oxidative damage that contributes to the exacerbation of DN. The klotho protein also has endogenous antifibrotic function via antagonism of Wnt/*β*-catenin signaling, suggesting that loss of *α*-klotho may contribute to the progression of DN by accelerated fibrogenesis [42]. In addition, suppressive effects of *α*-klotho on the insulin-like growth factor pathway may be associated with inhibitory action on renal fibrosis and cardio-renal protection in high oxidative stress conditions such as diabetes and its complications [42, 43].

Hence, the potential utility of the *α*-klotho-VDR axis in clinical practice is anticipated to be at least twofold. First, *α*-klotho has the potential to serve as an early and sensitive biomarker of DN. However, its specificity and its prognostic value and differential diagnostic value in human disease remain to be examined. Second, *α*-klotho exogenous supplementation and/or upregulation or restoration of *α*-klotho by reducing the iron levels via chelating agent or dietary iron restriction may provide novel therapy for DN patients with Hp 2-2 genotype to retard or block its progression to advanced CKD by arresting or slowing progression as well as by preventing and reversing complications [33].

It is important to note that the current study has some limitations. The prevalence of the Hp 2-2 genotype in Western societies including Israel is 35–50% while the prevalence of Hp 1-1 is only 10–15%. Furthermore, since the Hp 2-2 genotype is associated with the development of reduced GFR and increased development of diabetic complications, combined with the fact that the biopsies that we used in the study were taken only for clinical reasons, most of our DN patients have the Hp 2-2 genotype. Hence, we have a limited number of Hp 1-1 biopsies compared with Hp 2-2 biopsies. However, in this study, we explored our hypothesis by using the genetically engineered Hp 2-2 mouse model that represents the human DN, and we strengthened our observations with the human experiments. The STZ-induced type 1 diabetes C57B1/6 mouse has been widely used as a model for DN since this mouse model has the spectrum of micro- and macrovascular complications similar to human disease including hyper-filtration, glomerular hypertrophy, and albuminuria [13, 44].

## 5. Conclusion

**Figure 9:**
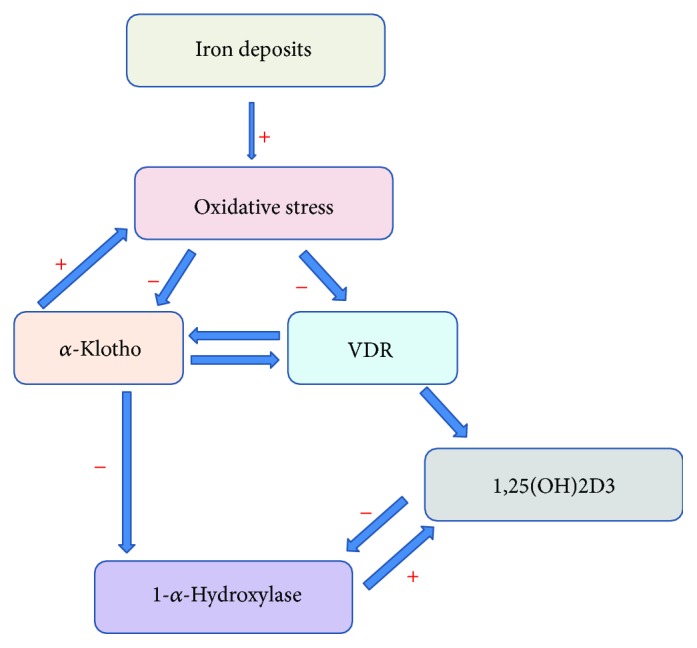
Suggested mechanism for the renal proximal convoluted tubule (PCT) injury in Hp 2-2 DN. The increased iron deposits in the PCT of the Hp 2-2 genotype generate a prooxidative environment in the kidney, leading to reduced renal expression of *α*-klotho and VDR proteins accompanied by an increased level of PCT injury. This subsequently interferes with vitamin D activation by 1-*α*-hydroxylase in the PCT, thereby increasing the kidney injury and the incidence of DN and its complications.

We conclude that the iron-klotho-VDR axis is a key player in the mechanism contributing to iron-mediated PCT injury in DN mice and patients with Hp 2-2 genotype. Based on our observations, we suggest a novel pathophysiological mechanism explaining why DN patients with Hp 2-2 genotypes suffer from severe renal PCT injury as well as why the progression to end-stage renal disease is increased in these patients. We propose that the increased iron deposits in the PCT of the Hp 2-2 genotype generate a prooxidative environment in the kidney, leading to reduced renal expression of *α*-klotho and VDR proteins, accompanied with a high level of PCT injury. It subsequently interferes with vitamin D activation by 1-*α*-hydroxylase in the PCT, thereby increasing the kidney injury and the incidence of DN or its complications among Hp 2-2 genotype patients (Figure 9). We believe that targeting the iron-klotho-vitamin D axis especially in DM Hp 2-2 patients may open the way for new ideas regarding the pathogenesis and treatment of early stages of DN [33].

## Figures and Tables

**Table 1:** Study participants' characteristics stratified by Hp phenotype.

	Hp 1-1	Hp 2-2
	*n* = 7	*n* = 35
Mean age (years) Range	48.88 ± 15.90 26–66	50.77 ± 13.55 21–67
Male/female (*n*) M/F ratio	4/3 1.33	25/10 2.5
DM/nonDM (*n*) % DM	1/6 14%	21/14 60%
Mean GFR Range	58.33 ± 21 29–84	48.57 ± 24 15–96
Mean systemic blood pressure	144/74 ± 20/13	141/72 ± 29/16

## Data Availability

The data used to support the findings of this study are available from the corresponding author upon request.
